# Evaluation of ammonia emission reducing effect by adding waste cooking oil in pilot-scale composting of dairy cattle manure

**DOI:** 10.5713/ab.23.0027

**Published:** 2023-05-04

**Authors:** Kazutaka Kuroda, Akihiro Tanaka, Kenichi Furuhashi, Naoki Fukuju

**Affiliations:** 1 Division of Livestock Research, Kyushu Okinawa Agricultural Research Center, National Agriculture and Food Research Organization, Suya, Koshi 861-1192, Japan; 2 Department of Biological and Environmental Engineering, Graduate School of Agricultural and Life Sciences, The University of Tokyo, Tokyo 113-8657, Japan

**Keywords:** Aeration Condition, Ammonia Emissions, Composting, Dairy Cattle Manure, Waste Cooking Oil

## Abstract

**Objective:**

In our previous study, we observed that the addition of waste cooking oil (WCO) reduced ammonia (NH_3_) emissions during laboratory-scale composting of dairy cattle manure under low-aeration condition. Therefore, this study aimed to evaluate the effect of addition of WCO on NH_3_ emissions reduction during pilot-scale composting of dairy cattle manure, which is close to the conditions of practical composting treatment.

**Methods:**

Composting tests were conducted using pilot-scale composting facilities (1.8 m^3^ of capacity). The composting mixtures were prepared from manure, sawdust, and WCO. Two treatments were set: without WCO (Control) and with WCO added to 3 wt% of manure (WCO3). Composting was conducted under continuous aeration at 40 L/min, corresponding to 22.2 L/(min·m^3^) of the mixture at the start of composting. The changes in temperatures, NH_3_ concentrations in the exhaust gases, and contents of the composted mixtures were analyzed. Based on these analysis results, the effect of WCO addition on NH_3_ emissions and nitrogen loss during composting was evaluated.

**Results:**

During composting, the temperature increase of the composting mixture became higher, and the decreases of weight and water content of the mixture became larger in WCO3 than in Control. In the decrease of weight, and the residual weight and water content of the mixture, significant differences (p<0.05) were detected between the two treatments at the end of composting. The NH_3_ concentrations in the exhaust gases tended to be lower in WCO3 than in Control. Nitrogen loss was 21.5% lower in WCO3 than in Control.

**Conclusion:**

Reduction of NH_3_ emissions by the addition of WCO under low aeration condition was observed in pilot-scale composting, as well as in laboratory-scale composting. This result suggests that this method is effective in reducing NH_3_ emissions in practical-scale composting.

## INTRODUCTION

Composting is commonly used in Japan to recycle animal manure intended to be used as a plant fertilizer [1]. From composting of manure, however, large amounts of malodorous gases, including concentrated ammonia (NH_3_), are emitted during the treatment process. It causes complaints against the malodor and various global environmental problems, such as acid rain and soil acidification [2,3]. Therefore, various countermeasures against malodor emissions from composting of animal manure have been studied and developed, in terms of both, the deodorization of emitted malodorous gases and the reduction of emissions themselves [3–5].

In recent years, several studies reported that NH _3_ emissions during composting of animal manure was remarkably reduced by adding cooking oil or waste cooking oil (WCO) to the composting of animal manure [6–8]. In our previous study [9], we evaluated the effect of adding WCO on NH_3_ emissions during laboratory-scale composting of dairy cattle manure. Under a low aeration condition corresponding to 21.4 L/(min·m^3^) of the initial composting mixture, a remarkable decrease in NH_3_ emissions was observed in the WCO-added treatments, to which WCO was added at lower than 3 wt% of manure, compared with the control treatment without WCO. In contrast, under the higher aeration condition corresponding to 37 L/(min·m^3^) of the initial mixture, NH_3_ emissions increased in the WCO-added treatments compared to the control treatment. These results demonstrate that the influence of WCO addition at a range below 3 wt% on NH_3_ emissions was affected by aeration condition during composting. This method should be considered for practical composting application.

In this study, the reduction in NH _3_ emissions during composting of dairy cattle manure by adding WCO was examined in pilot-scale composting tests closer to the conditions of practical composting treatment (1.8 m^3^ of volume and 900 to 950 kg of weight at the start of composting), and the effectiveness of this method was evaluated.

## MATERIALS AND METHODS

### Materials for composting

The materials for the composting were obtained as described previously [9]. Dairy cattle manure was collected from a dairy farmer at Kikuchi, Kumamoto Prefecture, Japan. Sawdust and WCO were purchased from a composting center (Koshi Bio X) in Koshi and Hayashi Sangyo Co. Ltd., Kumamoto, Japan, respectively.

### Composting test

**Figure 1:**
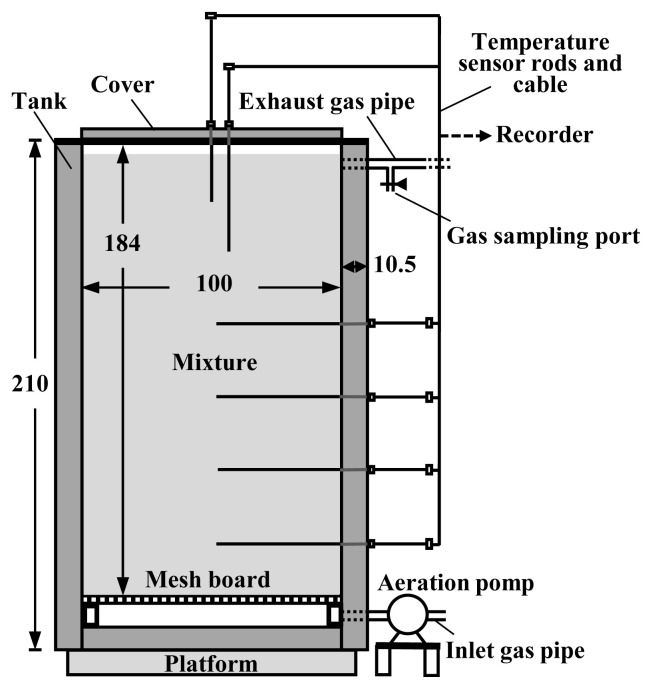
Scheme of composting facility. The unit of measures in the figure is cm.

The composting was conducted using pilot-scale composting facilities (Figure 1), the details of which are shown in Kuroda et al [10]. The main part of the facility was a tank with an inner capacity of 1.8 m^3^ (1 m in width and depth, 1.84 m in height), consisting of an outer shell (stainless-steel panel) and an inner filling for insulation (polystyrene board). The tank was filled with the composting material, and during composting, the material was continuously aerated using an air pump connected to the bottom of the tank. Thermocouple sensor rods were inserted into the tank to monitor the temperatures of the composting material during composting.

Table 1 lists the settings of the composting test. Composting mixtures were prepared from dairy cattle manure and sawdust by mixing a weight ratio of 5:1. Two treatments were set: WCO was added to the mixture at 0 wt% (Control) and 3 wt% (WCO3) of the manure. The mixtures were placed in the tanks and continuous aeration was supplied at a flow rate of 40 L/min, corresponding to 22.2 L/(min·m^3^) of the initial mixture in the tank at the start of composting. This aeration rate was close to the low aeration rate (21.4 L/(min·m^3^) of the initial mixture) at which reduction of NH_3_ emissions was observed after WCO addition in the laboratory-scale composting tests in our previous study [9]. It was substantially lower than those applied to practical-scale composting treatments of animal manure, 50 to 300 L/(min·m^3^) of the composting material, and usually ≥100 L/(min·m^3^) of the material [11].

The composting period was set to 28 d. During compost ing, exhaust gases were collected from the exhaust pipes connected near the top of the tank, and NH_3_ concentrations in the gases were measured using a detection tube (No. 3L or 3M; Gastec Co., Ayase, Japan) at intervals of 12 or 24 h. On days 7, 14, and 21, the mixtures were removed from the tanks, mixed completely, and placed in the tanks again (turning). At every turning, the mixtures were weighed before and after turning, and 3 kg of each mixture was collected after turning. The collected samples were analyzed using various analytical methods described below. Using the same settings, the composting test was repeated three times.

### Sample analyses

Analyses of the collected samples in the composting tests were conducted according to the study by Kuroda et al [10]. The water content, volatile solids (VS, roughly considered organic matter), and ash in the samples were analyzed by drying the samples at 105°C for two days followed by combustion at 550°C for 6 h. The extract of the sample was prepared using a 2 N KCl solution as shown in a previous report [12], and was used for measuring the pH.

Using the KCl extract solutions described above, ammonium nitrogen (NH_4_-N) and nitrite or nitrate nitrogen (NO_x_-N) in the samples were analyzed based on the method described by Bremner and Keeney [13]. Kjeldahl-nitrogen (Kj-N) was analyzed as described in our previous study [9] based on the methods of Bremner [14]. Organic nitrogen (ON) and total nitrogen (TN) were determined by subtracting NH_4_-N from Kj-N and summing Kj-N and NO_x_-N, respectively.

The water content and VS analyses were duplicated; the pH, NH_4_-N, and NO_x_-N analyses were performed in triplicate; and the Kj-N analysis was quintuplicated for each sample. From the analyzed values and the weights of the mixtures, the total contents of water, VS, ash, TN, ON, NH_4_-N, and NO_x_-N in the mixtures and nitrogen losses were calculated.

### Statistical analysis

The weight decreases, residual weights, and the contents of the composted mixture in Control and WCO3, measured or calculated as described above, were subjected to Student’s T test [15] at the start, 1st to 3rd turnings, and the end of composting.

## RESULTS

### Changes in the temperatures of the composted mixtures and NH_3_ emissions

The numerical data for the composting test shown below are the mean values of the repeated tests (n = 3).

**Figure 2:**
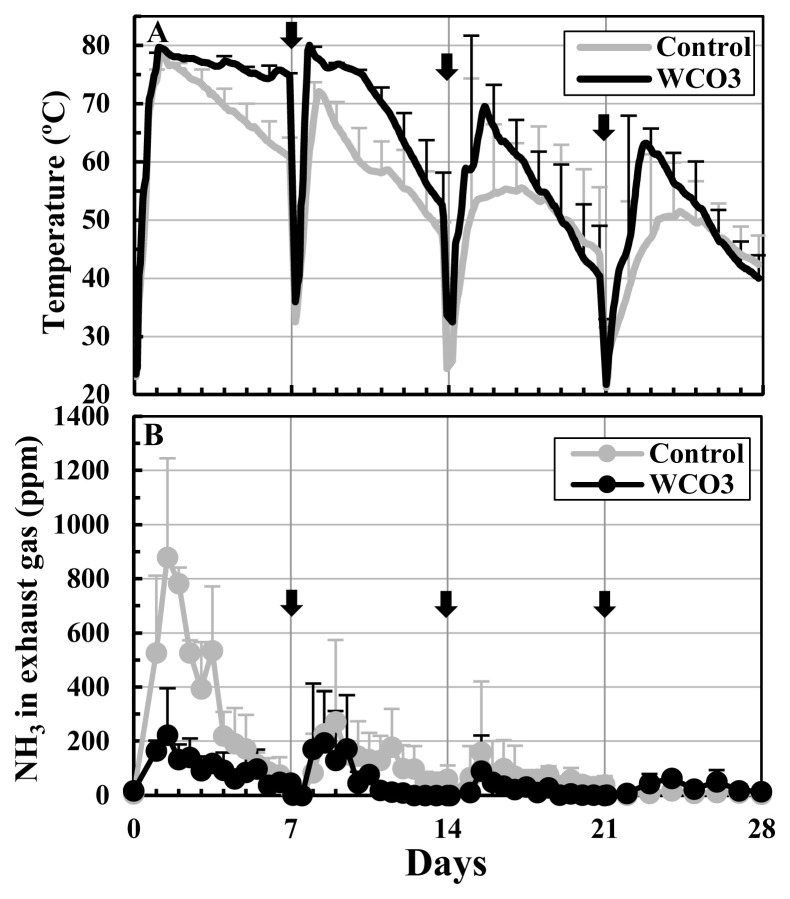
Changes in temperatures of the mixtures and NH_3_ concentrations in the exhaust gases during composting tests: (A) temperatures; (B) NH_3_ concentrations in the exhaust gases. The mean values at each measurement time point during repeated composting tests are plotted in the graphs. In the graph A, the temperatures at a height of 140 cm of the mixtures are plotted. The bars on the lines and symbols indicate standard deviations (n = 3). The arrows indicate turning.

Figure 2 shows the changes in the temperatures of the composted mixtures and NH_3_ concentrations in the exhaust gases from the composting facilities during the composting test. In both treatments, the temperatures rapidly increased to 80°C approximately one day after the start of the experiment, followed by a gradual decrease. After every turning, they repeated the increase and decrease, and after the 2nd turning (day 14), the degree of temperature increase declined with earlier temperature drop. The temperature in WCO3 was higher than that in Control during most of the composting period (Figure 2A). The NH_3_ concentrations in the exhaust gases remarkably increased within the first seven days, and during this period, the concentration in WCO3 was lower than that in Control. The concentrations peaked within 1.5 to 2.5 days from the start, and the peaks values were 880 ppm and 223 ppm in Control and WCO3, respectively, both at 1.5 day after the start of composting. Since the 2nd turning, the concentrations increased slightly, and since the 3rd turning (day 21), they remained below 100 ppm in both of the treatments to the end of composting (Figure 2B).

### Changes in the pH, weights, and the components of the composted mixtures

**Figure 3:**
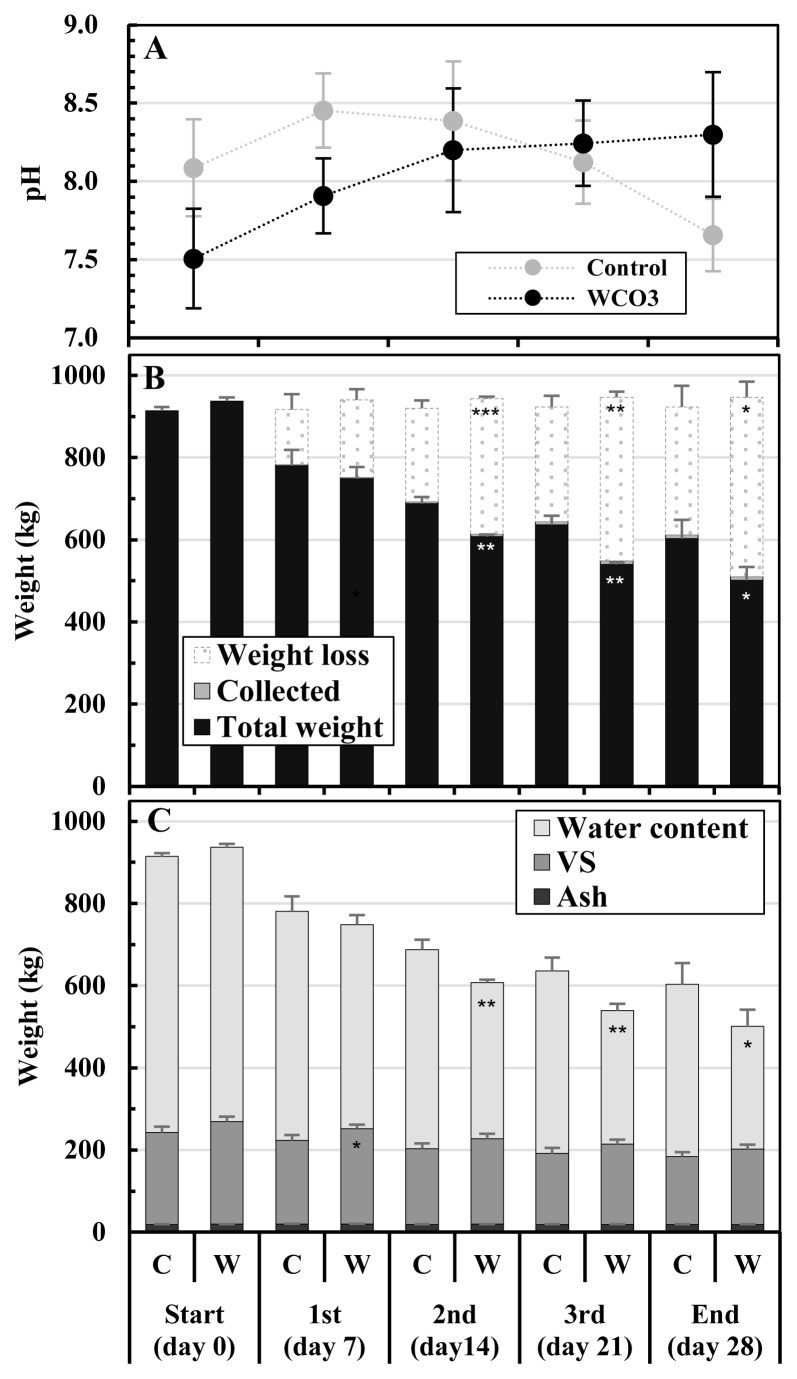
Changes in pH, weights, and components of the mixtures during composting test: (A) pH; (B) weights; (C) contents of the composted mixtures. The legends in graphs B and C are as follows: Total weight, the weight of the composted mixture; Collected, the weight of collected mixture; Weight loss, the weight decrease during composting; VS, volatile solids. The mean values at each measurement time point during repeated composting tests are plotted in the graphs. Bars on the symbols and respective parts of the columns indicate standard deviations (n = 3). On the bottommost horizontal axis, the characters C and W indicate two treatments, Control and WCO3, and 1st, 2nd, and 3rd indicate three turnings, as well as in Figure 4. Asterisks in the columns of WCO3 indicate significant differences between the values of corresponding items of Control and WCO3: * p<0.05; ** p<0.01; *** p<0.001.

Figure 3 shows the changes in pH, weights, and components of the mixtures during composting. The pH of the initial mixture was lower in WCO3 than in Control: 7.51 and 8.09, respectively. For WCO3, the pH gradually increased throughout the composting period and reached 8.30 at the end of composting. In contrast, the pH of Control increased to 8.45 by the 1st turning, and then decreased to 7.66 by the end (Figure 3A). In both the treatments, the weights of the mixtures decreased during composting, with the decreases occurring mainly (more than 70% of the total decreases) by the 2nd turning. In WCO3, the weight decrease of the mixture was larger and the weight of the mixture was smaller than that in Control, and since the 2nd turning, significant differences were detected between the two treatments. At the end of composting, the weights of the mixtures were 603.0 kg for Control and 501.3 kg for WCO3. The weights of collected mixtures (Collected) were very small and same in both treatments (9 kg in total during the test) (Figure 3B).

In the initial mixtures, the VS content was slightly higher in WCO3 (249.6 kg) than in Control (223.6 kg) by addition of WCO, and water and ash contents were generally the same in both treatments. The VS content gradually decreased during composting in both treatments, and the content in WCO3 was slightly larger than that in Control throughout composting, although a significant difference was only observed between those values at the 1st turning. At the end of composting, the VS contents were 165.0 kg in Control and 183.5 kg in WCO3. The water content in the initial mixtures were 671.9 kg in Control and 668.2 kg in WCO3, respectively. The water content decreased to 419.5 kg in Control, and 299.4 kg in WCO3 by the end of composting. Since the 2nd turning, the water content in WCO was significantly lower than that in Control. The ash contents decreased slightly in both the treatments by the end, due to the sampling of the mixtures at every turning (Figure 3C).

### Changes in nitrogen contents in the composted mixtures

**Figure 4:**
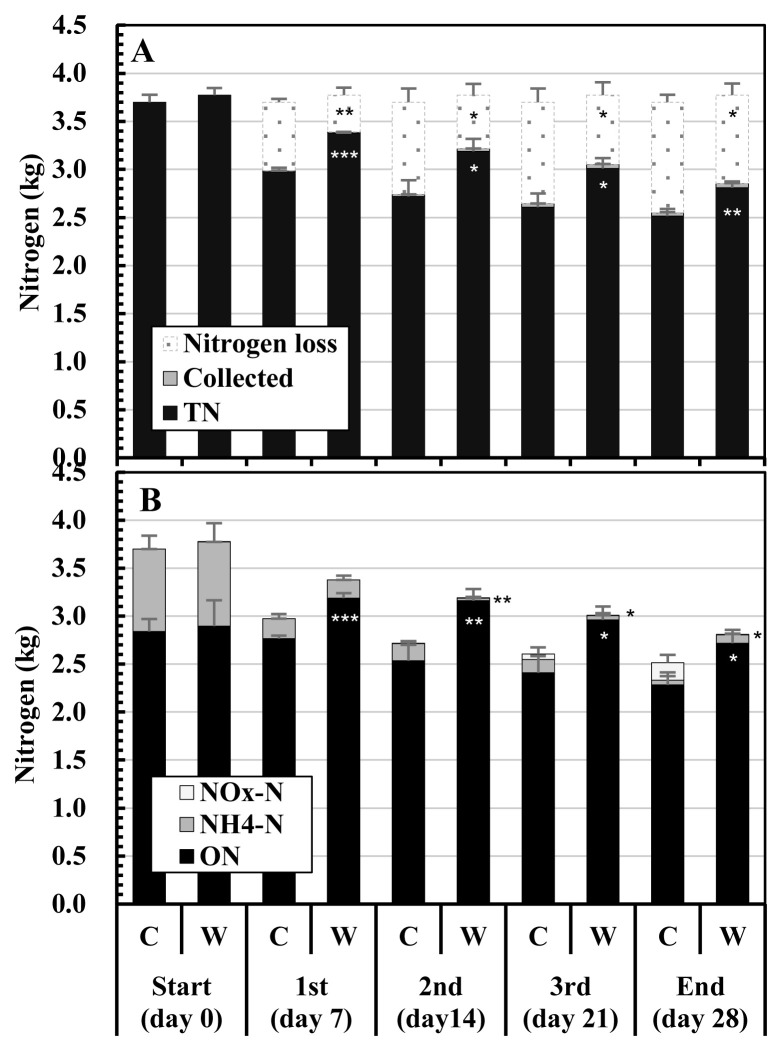
Changes in the nitrogen contents of the mixtures and nitrogen losses during composting test: (A) total nitrogen in the mixtures, nitrogen in the collected samples, and nitrogen losses; (B) nitrogen components in the mixtures. The legends in graphs (A) and (B) are as follows: Nitrogen loss, nitrogen loss during composting; Collected, nitrogen in the collected mixture; TN, total nitrogen in the mixture; NO_x_-N, nitrite or nitrate nitrogen; NH4-N, ammonium nitrogen; ON, organic nitrogen. The mean values at each measurement time point during repeated composting tests are plotted in the graphs. Bars on the respective parts of the columns indicate standard deviations (n = 3). Asterisks in the columns or on the right side of the columns of WCO3 indicate significant differences between the values of the corresponding items of Control and WCO3 at the respective periods: * p<0.05; ** p<0.01; *** p<0.001. The asterisks on the right side of the columns of WCO3 on days 14, 21, and 28 indicate significant differences between NH4-N contents (at days 14 and 21) and between NO_x_-N contents (at day 28) in the mixtures in Control and WCO3, respectively.

Figure 4 shows the changes in the nitrogen contents in the composted mixtures and nitrogen losses during composting. The TN contents in the initial mixtures were 3.70 kg in Control and 3.77 kg in WCO3, with no significant difference. The TN contents of the mixtures gradually decreased in both the treatments during composting. At the respective turnings and the end of composting, the TN content in the composted mixture was higher and the nitrogen loss was lower in WCO3 than in Control, and significant differences were detected between the two treatments (Figure 4A). At the end of composting, the TN contents in the mixtures and the nitrogen losses during composting were 2.52 kg and 1.15 kg in Control, and 2.81 kg and 0.92 kg in WCO3, respectively.

Figure 4B shows the changes in the nitrogen components (ON, NH_4_-N, and NO_x_-N) of the mixtures. In addition to TN, these components of the initial mixtures were similar in both treatments. The main nitrogen components in the initial mixtures were ON and NH_4_-N, and their content ratios to TN were similar in both treatments: ≈76.8% for ON and ≈23.2% for NH_4_-N. During composting, some differences were observed in the changes in the respective nitrogen components of the mixtures between the two treatments. The ON in Control gradually decreased throughout the composting period, whereas the ON in WCO3 increased by the 1st turning, and then gradually decreased by the end. From the 1st turning to the end, ON in WCO3 remained at a larger value than that in Control, and significant differences (p<0.001, 0.01, or 0.05) were detected between these values. NH_4_-N significantly decreased by the 1st turning in both the treatments, and afterwards, NH_4_-N in Control gradually decreased by the end. On the other hand, NH_4_-N in WCO3 almost disappeared by the 2nd turning, and then slightly increased by the end. Although NH_4_-N in WCO3 was significantly lower than that in Control at the 2nd turning (p< 0.001) and the 3rd turning (p<0.05), it was higher than that in Control at the end. NO_x_-N in Control accumulated from the 2nd turning, and it became higher than NH_4_-N by the end. In WCO3, NO_x_-N accumulated slightly since the 3rd turning, but was significantly lower than in Control (p<0.05) at the end. The nitrogen in the collected mixtures (Collected) were very small in both treatments (smaller than 0.05 kg in total during the test), and significant difference was not detected.

## DISCUSSION

Reduction of NH_3_ emissions from composting of animal manure by adding carbon sources, which promote the growth of microorganisms and the simultaneous assimilation of nitrogen, have been studied [16]. The reduction in NH_3_ emissions by the addition of WCO observed in previous studies [6,7,9] was presumed to be the effect of easily biodegradable lipids in WCO as the carbon source. In Japan, WCO discharged from business activities is classified as industrial waste, and the disposal or treatment of WCO is regulated by the Waste Management and Public Cleansing Law [17]. In accordance with this law, WCO collection systems work for reutilization in various regions [18,19]. Thus, WCO is considered a possible carbon source and an easily accessible material for reducing NH_3_ emissions from composting.

In the treatment WCO3 in this pilot-scale composting test, WCO addition (3 wt% of the manure) and low aeration rate (40 L/min, corresponding to 22.2 L/(min·m^3^) of the initial composting mixture) were applied. It followed the setting of the treatment with the same name ‘WCO3’ at the low aeration condition in the laboratory-scale composting test [9], in which reduction of NH_3_ emissions was observed. The addition of WCO resulted in a lower pH and larger VS in the initial mixture in WCO3 than in Control (Figures 3A, 3C), as well as in the laboratory-scale test [9]. During composting in the pilot-scale test, longer durations of higher temperatures were observed in the composted mixtures in both the treatments than in the laboratory-scale test. Additionally, a higher temperature of the mixture was observed in WCO3 than in Control during most of the composting period (Figure 2A). This could be caused by the larger heat generation from the active decomposition of added WCO, and it resulted in a larger decrease in total weight and water content in the mixture in WCO3 than in Control (Figures 3B, 3C). These trends were also observed in the laboratory-scale test [9] and other previous reports [7,20,21].

Concerning NH _3_ emissions, the NH_3_ concentrations in the exhaust gases were lower in WCO3 than in Control (Figures 2B), similar to the laboratory-scale test [9]. However, the changes in the respective nitrogen components in the mixtures during composting were different to some extent between the pilot-scale and laboratory-scale tests. In all the treatments with or without WCO in the laboratory-scale test, ON increased and NO_x_-N slightly accumulated, whereas NH_4_-N decreased but remained in a considerable amount (11.2% to 13.5% of TN in the initial mixtures) by the end of composting. In Control in the pilot-scale test, ON constantly decreased throughout the composting period and NH_4_-N almost disappeared by the end, whereas NO_x_-N increased from 2nd turning to the end of composting. In WCO3, ON increased only one time by the 1st turning, and decreased by the end of composting. NH_4_-N almost disappeared by the 2nd turning and slightly increased thereafter, whereas NO_x_-N slightly accumulated by the end (Figure 4B). These changes probably resulted in the changes in pH of the mixtures: decrease after the 1st turning in Control and constant increase by the end in WCO3 (Figure 3A). Seki and Oyanagi [21] observed a delay in nitrate-nitrogen accumulation in a composted mixture to which WCO was added, compared to the mixture without WCO during the composting of cattle manure. The addition of WCO might have led to preferential decomposition of WCO in the former stage of composting, resulting in a delay in the decomposition of the organic matter in the manure and prolonged NH_4_-N generation.

The ratios of nitrogen losses during composting to the TN contents in the initial mixtures were 31.0% in Control and 24.4% in WCO3 (Figure 4A), which were 2.5 to 2.6 times larger than those in the laboratory-scale test (12.3% in Control and 9.4% in WCO3). These larger nitrogen losses could be the result of larger NH_3_ emissions caused by longer durations of higher temperatures during composting, as mentioned above. Assuming that the loss in Control corresponded to 100%, the loss in WCO3 was 78.5%, which was 21.5% lower than in Control.

Except for the low aeration rate, the conditions of the composting tests in this study (1.8 m height of piled material and aeration from the bottom of the pile) were close to those in the practical composting facilities with the forced aeration system. The results of this study suggest that the combination of WCO addition and supplementation with low-aeration condition is also effective for reducing NH_3_ emissions in practical composting treatments. Moreover, evaluation of the effectiveness of this method in composting under low aeration conditions close to natural ventilation in the piled material and evaluation of prepared compost as a plant fertilizer should be examined in the future.

## Figures and Tables

**Table 1:** Composting test parameters

Treatments	Materials (kg)			Initial mixtures (kg)^1)^

	Manure	Sawdust	WCO	
Control	820	164	-	914.0, 905.5, 923.5
WCO3	820	164	24.6 (3.0)^2)^	937.0, 928.0, 946.5

WCO, waste cooking oil.

1) The weights of the mixtures in the two treatments placed in the composting facilities at the beginning of the composting tests are indicated in sequence of repeated tests. These were different in each test because the bulk densities of the mixtures varied to a certain extent in each test. Considering the added amount of WCO, the weights of WCO3 were set at 22.5 or 23.0 kg larger than those of Control.

2) The value in parentheses is the weight percent of WCO to dairy cattle manure.
